# Deletion of exocyst component 5 suppresses repair of injured kidney by limiting cell proliferation

**DOI:** 10.1038/s41420-026-03127-6

**Published:** 2026-04-24

**Authors:** Hui Jae Lim, Min Jung Kong, Mira Noh, You Ri Park, Yong Kwon Han, Joshua H. Lipschutz, Kwon Moo Park

**Affiliations:** 1 Department of Biomedical Science and BK21 Plus, The Graduate School of Kyungpook National University, Daegu, Republic of Korea; 2 Department of Anatomy, School of Medicine, Kyungpook National University, Daegu, Republic of Korea; 3 Department of Anatomy, Cardiovascular Research Institute, School of Medicine, Kyungpook National University, Daegu, Republic of Korea; 4 Department of Medicine, Medical University of South Carolina and Ralph H. Johnson Veterans Affairs Medical Center, Charleston, SC USA

**Keywords:** Cell division, Chronic kidney disease, Nephrons, Experimental models of disease, Renal fibrosis

## Abstract

Impaired cell proliferation causes fibrotic changes in tissues, leading to loss of function. Although exocyst component 5 (Exoc5), a central component of the eight-protein exocyst complex, regulates the targeting and docking of intracellular vesicles which are essential for cell proliferation, its role in tissue regeneration remains to be defined. Here, we investigated the role of Exoc5 in the repair of kidney injury induced by ischemia-reperfusion (I/R) using proximal tubule cell (PTC)-specific Exoc5 knockout (Exoc5^KO^) mice generated by crossing *Exoc5*^*f/f*^ with *PEPCK-cre* mice. Exoc5^KO^ and wild-type (Exoc5^WT^) mice were subjected to either bilateral kidney I/R or sham surgery. I/R induced functional and structural kidney damage in both Exoc5^KO^ and Exoc5^WT^ mice, as evidenced by increased plasma creatinine and BUN, decreased glomerular filtration rate, and histological damage. Kidney function and structure gradually improved in both Exoc5^KO^ and Exoc5^WT^ mice over time; however, neither group fully recovered normal function, and the recovery was less pronounced in Exoc5^KO^ than in Exoc5^WT^ mice. Twenty-one days after I/R, Exoc5^KO^ mice showed greater collagen deposition and α-smooth muscle actin (α-SMA) and vimentin expression compared to Exoc5^WT^ mice, whereas E-cadherin expression was lower. Post-I/R PTC proliferation in Exoc5^KO^ mice was significantly lower than in Exoc5^WT^ mice. In contrast, post-I/R induction of paired box 2 (Pax2) was greater in Exoc5^KO^ than in Exoc5^WT^ mice. In HK-2 cells, a human PTC line, Exoc5 downregulation by siRNA increased Pax2 expression and further increased N-cadherin, phosphorylated-Smad3 (p-Smad3), and α-SMA expression compared to control cells following TGF-β treatment. Collectively, these findings indicate that loss of Exoc5 impairs PTC regeneration and exacerbates fibrosis in the injured kidney, suggesting its therapeutic potential in preventing the transition from acute kidney injury (AKI) to chronic kidney disease (CKD).

## Introduction

Proliferation of surviving parenchymal cells following injury restores cell numbers and re-establishes proper tissue architecture, with subsequent differentiation ensuring recovery of specialized cellular functions [1]. Delayed or aberrant proliferation and differentiation leads to progressive structural deterioration, eventually resulting in organ dysfunction and fibrosis [2, 3]. Hence, understanding the molecular mechanisms that govern cell cycle re-entry, proliferation, and differentiation is essential to developing strategies for repair without promoting fibrotic remodeling. Despite significant advances in understanding the mechanisms of fibrosis, effective antifibrotic strategies have yet to be developed.

The exocyst, a highly conserved octameric protein trafficking complex, composed of exocyst components 1 to 8 (Exoc1-8), functions as a key mediator in the targeting and docking of post-Golgi secretory vesicles to specific sites on the plasma membrane [4, 5]. Among the exocyst components, Exoc5 (also known as Sec10) is the central linker which binds Exoc6, which, in turn, binds the GTP form of Rab8 found on the targeted post-Golgi vesicles, and the rest of the exocyst complex located at the specific site on the plasma membrane [6]. Studies have demonstrated that Exoc5 is essential for the function and development of various organs, including the kidney [7–9]. Exoc5 deletion in podocytes and urothelial cells results in spontaneous foot process effacement and ureteral obstruction, respectively, both of which lead to early death in mice [7, 8]. In addition, our previous in vitro studies using cultured renal epithelial cells revealed that Exoc5 regulates cell migration, polarity formation, and resistance to oxidative stress, all of which are critical for the repair of damaged organs [10–13]. These findings suggest that Exoc5 is critical for proper repair of tissues following injury. However, its role in the process of damaged tissue repair remains to be defined.

Ischemia/reperfusion (I/R) injury is common in various clinical settings, including transplantation and surgery. In the kidney, I/R, a major cause of acute kidney injury (AKI), leads to the loss of tubular epithelial cell polarity, cell death, de- and re-differentiation [14]. The kidney has substantial regenerative capacity, which, if properly managed, enables recovery after I/R injury. However, in the absence of appropriate treatments or when normal repair processes are impaired, I/R-induced AKI progresses to chronic kidney disease (CKD) and, ultimately, end-stage kidney disease (ESKD), both of which are serious clinical problems [15–17]. Considering that Exoc5 regulates processes involved in organ repair, we hypothesized that Exoc5 is a key regulator of kidney injury and subsequent repair. Here, we investigated the role of Exoc5 in the repair process after I/R injury using proximal tubule cell (PTC)-specific Exoc5 knockout (Exoc5^KO^) mice.

## Results

### Exoc5 deletion impairs the recovery of kidneys following ischemia/reperfusion injury

**Fig. 1:**
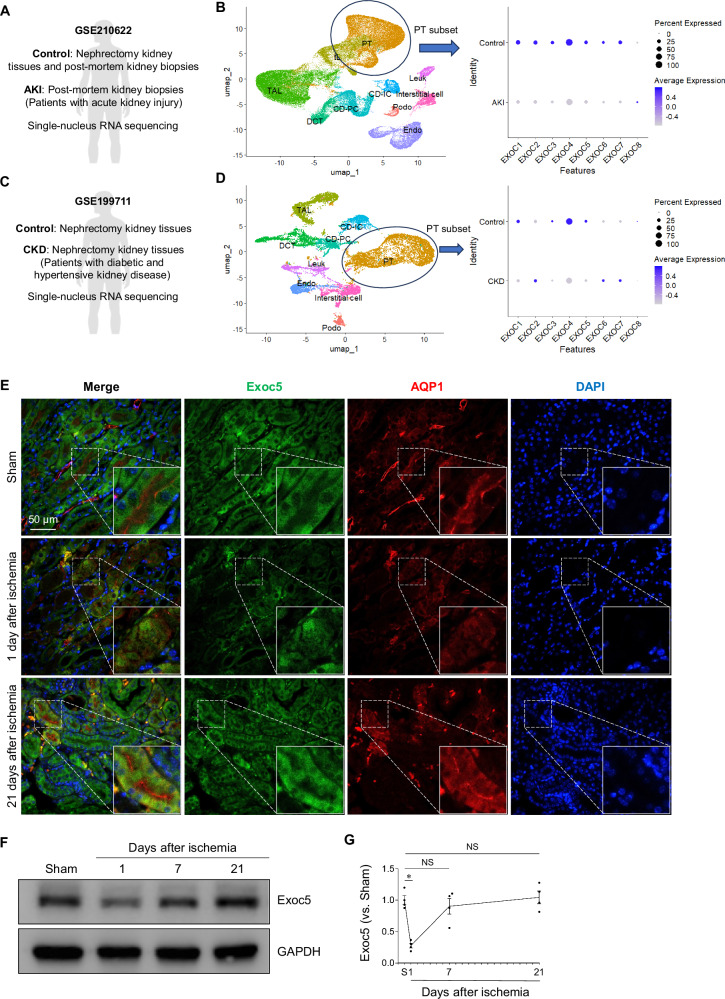
Expression of Exoc5 in kidneys of patients with AKI or CKD and in kidneys of mice subjected to kidney ischemia-reperfusion (I/R). **A**–**D** snRNA-seq data of the kidneys from patients with AKI and CKD were obtained from the Gene Expression Omnibus (GEO) database (GSE210622 for AKI and GSE199711 for CKD). **A**, **C** Schematic information of the snRNA-seq datasets. **B**, **D** Uniform Manifold Approximation and Projection (UMAP) visualization from the AKI and CKD dataset showing major kidney cell populations. Dot plot showing the expression of exocyst complex components (*EXOC1–8*) in proximal tubule cell (PT) cluster. Dot size represents the percentage of cells expressing the gene, and color intensity indicates the average expression level. **E**–**G** Wild-type mice were subjected to either 25 min of bilateral renal ischemia-reperfusion (I/R) or a sham surgery (sham). **E** Kidney sections were immunofluorescence-stained with antibodies against Exoc5 (green) and AQP1 (red); DAPI (blue) was used to visualize nuclei. **F**, **G** Exoc5 levels in kidneys were analyzed by Western blot, with GAPDH as the loading control; band densities were quantified using ImageJ (*n* = 4). Results are expressed as mean ± SEM. S, Sham; *, *p* < 0.05; NS not significant.

First, to determine whether EXOC5 is associated with AKI and CKD, we analyzed EXOC5 expression in kidneys from patients with AKI and CKD using single-nucleus RNA sequencing (snRNA-seq) datasets available in the Gene Expression Omnibus (GEO) database (Fig. 1A–D). In the snRNA-seq analysis, we observed a reduction in the expression of most exocyst complex components (*EXOC1* to *EXOC7*, except *EXOC8*) in PTCs from patients with AKI, and a reduction in *EXOC1*, *EXOC3*, *EXOC4*, and *EXOC5* expression in PTCs from patients with CKD (Fig. 1B, D). These results indicate that AKI and CKD are associated with decreased expression of exocyst components, including EXOC5.

Next, we assessed Exoc5 expression in the kidneys of wild-type (Exoc5^WT^) mice 24 h and 21 days after 25 min of bilateral kidney ischemia or sham operation. In sham-operated kidneys, Exoc5 was expressed throughout renal tubular epithelial cells, including aquaporin-1 (AQP1) positive cells, with AQP1 being a marker of proximal tubule cells, PTCs (Fig. 1E). Exoc5 expression in the majority of tubular cells was reduced 24 h after I/R compared with sham-operated controls and returned to near-sham levels by day 21 post-I/R (Fig. 1E). Consistent with histological data, the amount of Exoc5 expression in whole-kidney lysates significantly decreased 24 h after ischemia, and gradually returned toward sham levels by day 21 post-I/R (Fig. 1F, G).

**Fig. 2:**
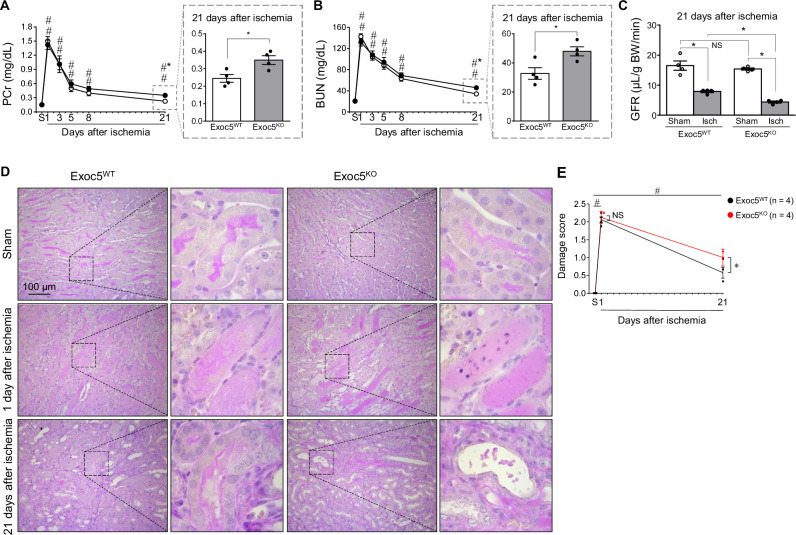
Inhibition of kidney repair by Exoc5 deletion following kidney ischemia-reperfusion (I/R). Exoc5^WT^ and Exoc5^KO^ mice were subjected to either 25 min of bilateral renal ischemia-reperfusion (I/R) or a sham surgery (sham). **A**–**C** Plasma creatinine (PCr), blood urea nitrogen (BUN), and creatinine clearance (CrCl) were measured in mice. **D**, **E** Kidney sections were PAS-stained, and kidney damage was scored as previously described. Results are expressed as mean ± SEM (*n* = 4). S Sham, ND not detected; #, *p* < 0.05 versus respective sham-operated mice; *, *p* < 0.05 versus ischemia-operated Exoc5^WT^ mice; NS not significant.

Given that Exoc5 expression was reduced in human AKI and CKD kidneys and in post-I/R mouse kidneys, we examined whether Exoc5 deletion affects I/R-induced kidney injury and repair using PTC-specific Exoc5-deleted (Exoc5^KO^) and wild-type (Exoc5^WT^) mice. PTCs are a major cell type in the kidney and are most susceptible to I/R injury [16]. I/R significantly increased plasma creatinine (PCr) and blood urea nitrogen (BUN) levels in both groups, peaking at 24 h post-I/R (Fig. 2A, B). However, PCr and BUN levels did not differ between Exoc5^KO^ and Exoc5^WT^ mice at 24 h post-I/R (Fig. 2A, B). Over time, PCr and BUN levels gradually decreased in both groups by day 21 post-I/R, but these levels remained higher in both groups than in the respective sham-operated mice (Fig. 2A, B); PCr and BUN levels were significantly higher in Exoc5^KO^ than in Exoc5^WT^ mice at day 21 post-I/R (Fig. 2A, B). In addition, glomerular filtration rates (GFR) were significantly decreased in both Exoc5^KO^ and Exoc5^WT^ mice at day 21 post-I/R compared with their respective sham controls, with a greater decline in Exoc5^KO^ than in Exoc5^WT^ mice (Fig. 2C). These data indicate that Exoc5 deletion hinders functional recovery following kidney I/R injury. Consistent with kidney functional parameters, kidneys from both groups showed severe tubular injury at 24 h post-I/R, with no significant between-group differences (Fig. 2D, E). Despite partial structural recovery by day 21 post-I/R, both groups still exhibited abnormal renal lesions, including tubular flattening and congestion, increased interstitial cell infiltration, and interstitial matrix expansion, indicating kidney fibrosis (Fig. 2D, E). These lesions were more severe in Exoc5^KO^ than in Exoc5^WT^ mice (Fig. 2D, E). These data indicate that, although Exoc5 deletion does not affect early susceptibility to I/R injury, it impairs the recovery process following I/R.

### Exoc5 deletion augments I/R-induced fibrosis

**Fig. 3:**
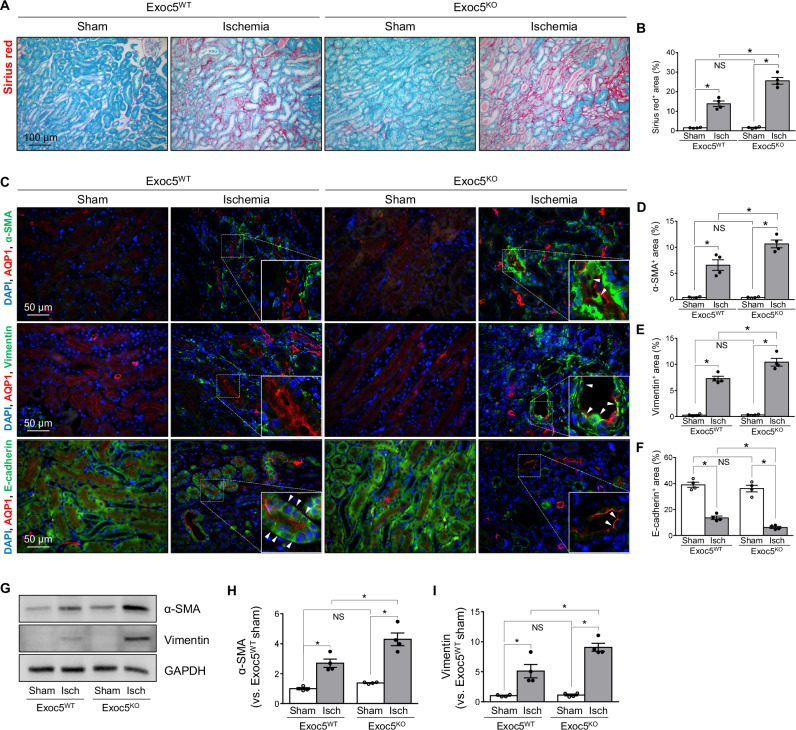
Exacerbation of kidney fibrosis by Exoc5 deletion following I/R. Exoc5^WT^ and Exoc5^KO^ mice were subjected to either 25 min of bilateral renal ischemia-reperfusion (I/R) or sham surgery (sham). **A**–**F** Kidney sections were stained with picrosirius red and immunofluorescence-stained with antibodies against α-SMA, vimentin, E-cadherin, and AQP1; DAPI was used to visualize nuclei. α-SMA- and vimentin-positive cells were observed in AQP1-positive tubules (arrowheads in **C**, top and middle panels). E-cadherin expression was not restored and was weak in AQP1-positive tubules (arrowheads in **C**, bottom panels). Collagen deposition and α-SMA-, vimentin-, and E-cadherin-positive areas were quantified using ImageJ. **G**–**I** α-SMA and vimentin levels in kidneys were analyzed by Western blot, with GAPDH as the loading control; band densities were quantified using ImageJ. Results are expressed as mean ± SEM (*n* = 4). *, *p* < 0.05; NS not significant.

Since aberrant kidney repair progresses to kidney fibrosis [3, 17], we assessed whether Exoc5 deletion affects kidney fibrosis after I/R. I/R increased picrosirius red-positive areas in kidneys from both groups, and these increases were greater in Exoc5^KO^ than in Exoc5^WT^ mice at day 21 post-I/R (Fig. 3A, B). In addition, I/R increased α-smooth muscle actin (α-SMA, a component of the stress fibers of myofibroblasts) and vimentin (a type III intermediate filament protein in fibroblasts and mesenchymal cells) expression in the interstitium of both Exoc5^KO^ and Exoc5^WT^ mice, whereas it decreased E-cadherin (epithelial cell polarity-maintaining protein) expression (Fig. 3C–F). These I/R-induced changes were more pronounced in Exoc5^KO^ than in Exoc5^WT^ mice (Fig. 3C–F). Consistent with histological data, I/R increased the amount of α-SMA and vimentin expression in both groups at day 21 post-I/R, and these increases were greater in Exoc5^KO^ than in Exoc5^WT^ mice (Fig. 3G–I). These data indicate that Exoc5 deletion exacerbates kidney fibrosis following I/R.

### Exoc5 deletion impairs cell proliferation after kidney I/R injury

**Fig. 4:**
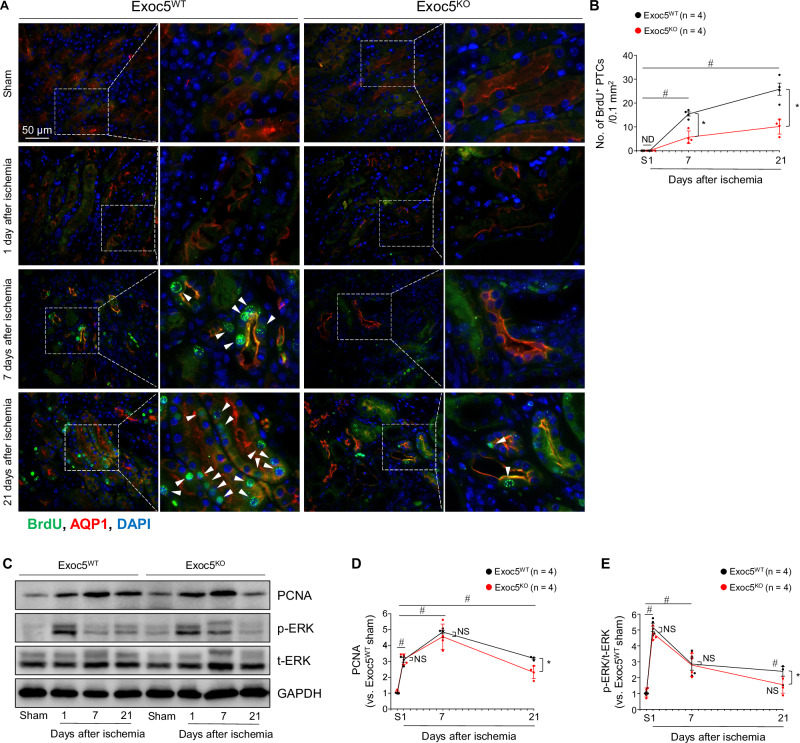
Inhibition of cell proliferation by Exoc5 deletion following I/R. Exoc5^WT^ and Exoc5^KO^ mice were subjected to either 25 min of bilateral renal ischemia-reperfusion (I/R) or sham surgery (sham). **A**, **B** Kidney sections were immunofluorescence-stained with antibodies against BrdU and AQP1; DAPI was used to visualize nuclei. Arrowheads indicate BrdU-incorporated proximal tubule cells. BrdU-incorporated cells were counted in proximal tubules in 40× micrographs (field area, 0.1 mm^2^). **C**–**E** PCNA, p-ERK, and t-ERK levels in kidneys were analyzed by Western blot, with GAPDH as the loading control; band densities were quantified using ImageJ. Results are expressed as mean ± SEM (*n* = 4). S Sham, ND not detected; #, *p* < 0.05 versus respective sham-operated mice; *, *p* < 0.05 versus ischemia-operated Exoc5^WT^ mice; NS not significant.

To determine whether delayed repair and exacerbated fibrosis after I/R injury observed in Exoc5^KO^ mice is associated with reduced proliferation, we administered 5-bromo-2’-deoxyuridine (BrdU) to mice every other day until sacrifice and quantified BrdU incorporation. BrdU is incorporated into DNA during replication in the S phase of the cell cycle [18]. BrdU-incorporated PTCs were detected in both Exoc5^KO^ and Exoc5^WT^ mice at 7 and 21 days post-I/R (Fig. 4A, B). The number of BrdU-incorporated PTCs was significantly lower in Exoc5^KO^ than in Exoc5^WT^ mice at 7 and 21 days post-I/R. BrdU-incorporated PTCs were not detected in either group after sham operation or at 24 h post-I/R (Fig. 4A, B). The expression of proliferating cell nuclear antigen (PCNA), which is expressed during cell proliferation and is a cofactor for DNA polymerases [19], reached a peak at 7 days post-I/R in both groups and then decreased by 21 days post-I/R (Fig. 4C, D). PCNA levels at 21 days post-I/R in Exoc5^KO^ mice were significantly lower than in Exoc5^WT^ mice (Fig. 4C, D). Phosphorylation of extracellular signal-regulated kinase (p-ERK/t-ERK), representing activation of a key component of the mitogen-activated protein kinase (MAPK) pathway regulating cell proliferation [20], peaked at 24 h post-I/R and then gradually decreased over time (Fig. 4C, E). ERK phosphorylation was lower in Exoc5^KO^ than in Exoc5^WT^ mice at 21 days post-I/R (Fig. 4C, E). These data indicate that Exoc5 deletion inhibits the regeneration of PTCs following kidney I/R.

### Exoc5 deletion augments post-I/R Pax2 expression

**Fig. 5:**
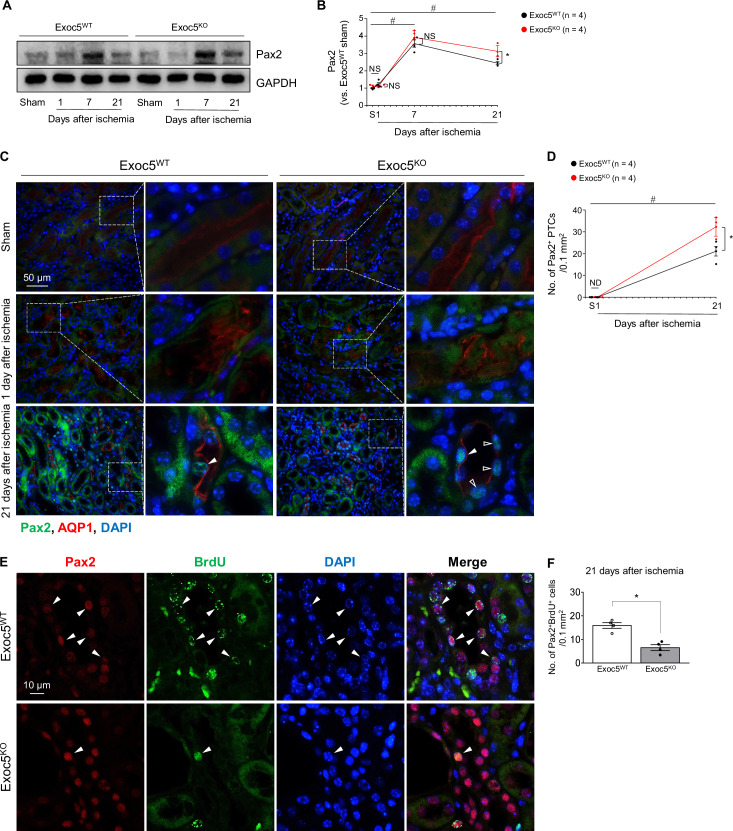
Increased Pax2 re-expression by Exoc5 deletion following I/R. Exoc5^WT^ and Exoc5^KO^ mice were subjected to either 25 min of bilateral renal ischemia-reperfusion (I/R) or sham surgery (sham). **A**, **B** Pax2 levels in kidneys were analyzed by Western blot, with GAPDH as the loading control; band densities were quantified using ImageJ. **C**, **D** Kidney sections were immunofluorescence-stained with antibodies against Pax2 and AQP1; DAPI was used to visualize nuclei. Filled arrowheads indicate Pax2-positive cells that express AQP1. Open arrowheads indicate Pax2-positive cells that do not express AQP1. Pax2-positive proximal tubule cells were counted in tubules in 40× micrographs (field area, 0.1 mm^2^). **E**, **F** Kidney sections were immunofluorescence-stained with antibodies against Pax2 and BrdU; DAPI was used to visualize nuclei. Arrowheads indicate Pax2- and BrdU-double-positive cells. Pax2- and BrdU-double-positive cells were counted in tubules in 40× micrographs (field area, 0.1 mm^2^). Results are expressed as mean ± SEM (*n* = 4). S Sham, ND not detected; #, *p* < 0.05 versus respective sham-operated mice; *, *p* < 0.05 versus ischemia-operated Exoc5^WT^ mice; NS not significant.

Paired box 2 (Pax2), a paired box family nuclear transcription factor, is essential for conversion of metanephric mesenchymal cells into epithelium during kidney development and is nearly absent in PTCs after nephron maturation [21–23]. However, Pax2 is re-expressed in injured or de-differentiated PTCs in pathological conditions such as fibrosis [24–26]. Damaged PTCs undergo de-differentiation and re-express Pax2 during kidney repair [27]. Therefore, we evaluated whether impaired kidney recovery and proliferation in the Exoc5^KO^ mice were associated with Pax2 expression. At 7 and 21 days post-I/R, Pax2 expression increased in both groups compared to their respective sham controls and was significantly higher in Exoc5^KO^ mice compared to Exoc5^WT^ mice at 21 days post-I/R (Fig. 5A, B). Consistent with the Western blot data, at 21 days post-I/R, nuclear Pax2-positive PTCs were detected in both groups, and their number was significantly higher in Exoc5^KO^ compared to Exoc5^WT^ mice (Fig. 5C, D). In cells expressing Pax2, a loss of typical localization patterns of AQP1, which is normally localized to the apical membrane in PTCs, was observed (Fig. 5C, indicated by open arrowheads).The levels of Pax2 expression at 24 h after I/R were similar to that in sham-operated mice in both groups (Fig. 5A, B). Also, Pax2-positive PTCs were not detected in either group after sham surgery or at 24 h after I/R (Fig. 5C, D). These data indicate that I/R stimulates Pax2 re-expression in PTCs during kidney fibrosis and Exoc5 deletion enhances Pax2 re-expression.

Since Pax2 re-expression in PTCs can contribute to kidney repair through proliferation, we examined whether Pax2 affects the differentiation of proliferated proximal tubule cells by the double-staining of Pax2 and BrdU. At 21 days post-I/R, although the number of Pax2-postive cells were greater in the in Exoc5^KO^ than Exoc5^WT^ mice (Fig. 5C, D), the number of Pax2 and BrdU double-positive PTCs was lower in Exoc5^KO^ compared with Exoc5^WT^ mice (Fig. 5E, F). These results suggest that increased Pax2 is more likely to induce the differentiation of newly formed cells into mesenchymal cells rather than into epithelial cells. These data indicate that Exoc5 deletion impedes maturation of regenerated tubule cells, consequently causing abnormal differentiation of tubular epithelial cells.

### Exoc5 knockdown increases Pax2 expression and augments TGF-β-induced EMT in human PTCs

**Fig. 6:**
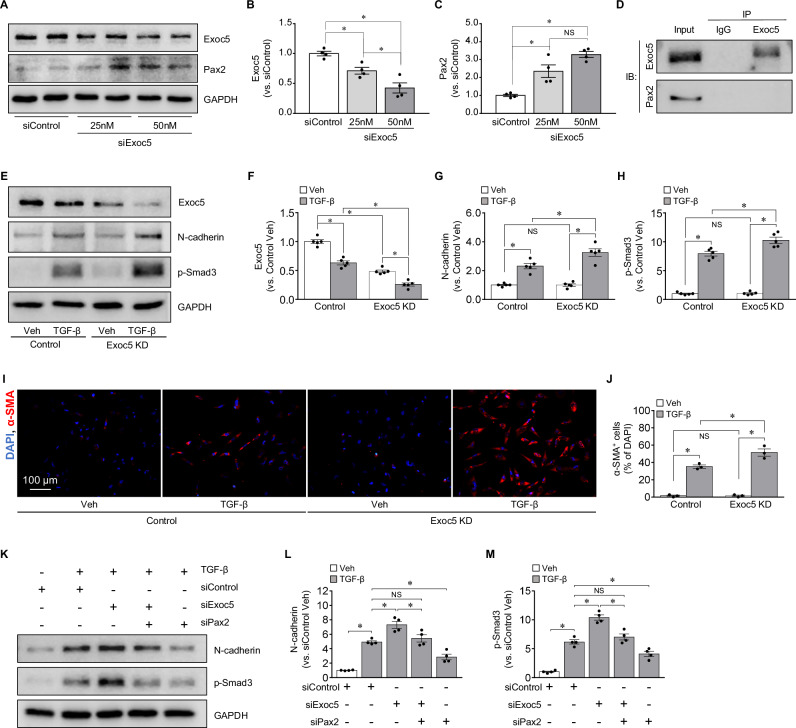
Pax2 expression and EMT progression in Exoc5-siRNA-treated HK-2 cells after TGF-β treatment. HK-2 cells were transfected with either Exoc5-siRNA or scrambled siRNA and then treated with or without 5 ng/mL TGF-β for 48 h. **A**–**H** Exoc5, Pax2, N-cadherin, p-Smad3 levels in cell lysates were analyzed by Western blot, with GAPDH as the loading control. **D** Co-immunoprecipitation was performed using an anti-Exoc5 antibody or normal IgG in HK-2 cell lysates, followed by Western blot analysis for Exoc5 and Pax2. **I**, **J** Fixed cells were immunofluorescence-stained with an anti-α-SMA antibody and α-SMA-positive cells were counted in a 20x micrograph; DAPI was used to visualize nuclei. **K**–**M** N-cadherin and p-Smad3 levels in cell lysates were analyzed by Western blot, with GAPDH as the loading control; band densities were quantified using ImageJ. Results are expressed as mean ± SEM (*n* = 3–5). *, *p* < 0.05; NS not significant.

Finally, we determined whether siRNA-mediated downregulation of Exoc5 affects epithelial-to-mesenchymal transition (EMT) in HK-2 cells, a human PTC line. To induce EMT, HK-2 cells were exposed to TGF-β for 48 h. First, we confirmed that Exoc5 siRNA (Exoc5 knockdown, Exoc5 KD) significantly decreased Exoc5 expression compared with scrambled siRNA (control) (Fig. 6A, B). In addition, Exoc5 downregulation increased Pax2 expression compared to control cells (Fig. 6A, C). These results indicate that Exoc5 downregulation leads to increased Pax2 expression.

To further define whether Exoc5 and Pax2 interact, we conducted co-immunoprecipitation (Co-IP) using an Exoc5 antibody. In these Co-IP experiments, Pax2 was not detected in the Exoc5 immunoprecipitates (Fig. 6D), indicating that Exoc5 does not directly bind to Pax2.

Next, we determined whether Exoc5 knockdown affects TGF-β-induced EMT. TGF-β treatment decreased Exoc5 expression in both control and EXOC5 KD cells (Fig. 6E, F). TGF-β treatment also increased the expression of N-cadherin and phosphorylated-Smad3 (p-Smad3) and α-SMA-positive cells in both Exoc5 KD and control cells, and these increases were greater in Exoc5 KD compared with control cells (Fig. 6E–H). Exoc5 KD alone did not affect the expression of N-cadherin and p-Smad3 (Fig. 6E–H). To examine whether exacerbation of TGF-β-induced EMT in Exoc5 KD is associated with increased Pax2 expression, we evaluated that Pax2 downregulation affects EMT enhanced by Exoc5 downregulation. Pax2 knockdown alone attenuated TGF-β-induced increases in N-cadherin and p-Smad3 expression (Fig. 6K–M and Supplementary Fig. 3D–F). Moreover, simultaneous knockdown of Exoc5 and Pax2 prevented the Exoc5 knockdown-mediated exacerbation of TGF-β-induced increases in N-cadherin and p-Smad3 expression (Fig. 6K–M). Pax2 siRNA decreased Pax2 expression without significant alteration of Exoc5 expression compared with control cells (Supplementary Fig. 3A–C). These results indicate that Exoc5 downregulation exacerbates TGF-β-induced EMT and this exacerbation is associated with increased Pax2 expression.

## Discussion

Repair of damaged organs proceeds through regeneration, in which tissues return to their pre-injury state, or through fibrosis, which may involve both regeneration of damaged cells and transformation of original cells into myofibroblasts. Typical features of inappropriate repair of damaged epithelial cells include loss of polarity and acquisition of mesenchymal characteristics, which together promote fibrosis. Evidence indicates that maladaptive repair is a complex interplay of multiple factors, including cell-cycle arrest, sustained oxidative stress, apoptosis, and loss of cell polarity, ultimately leading to fibrosis [3, 16, 17, 28]. Renal tubular epithelial cells have the ability to regenerate and restore kidney function following AKI [29]. In the S3 segment of the proximal tubule, which is the most vulnerable to ischemic AKI, of developing rat kidneys about half of the cells express markers for the G1-phase rather than quiescent G0-phase for immediate division if proliferation urgently required [30, 31]. It has been known that injured or regenerating cells transiently acquire mesenchymal characteristics to proliferate or migrate after injury and then re-differentiate into mature tubule cells [31]. Failure to restore epithelial characteristics during this process delays recovery and leads to organ dysfunction and fibrosis. Thus, the capacity of PTCs to re-enter the cell cycle and complete epithelial re-differentiation is critical for kidney repair and prevention of the AKI-to-CKD transition.

In the present study, Exoc5 deletion suppressed PTC proliferation. In addition, Exoc5 deletion reduced PCNA expression and ERK phosphorylation, key mediators of DNA synthesis and mitogenic signaling during renal epithelial proliferation in kidney repair, at 21 days post-I/R. Furthermore, Exoc5 deletion inhibited PTC differentiation prolonging the undifferentiated status at 21 days post-I/R as indicated by increased vimentin and reduced E-cadherin expression in PTCs. Therefore, we speculate that Exoc5 is required for the repair process in injured kidneys. Supporting our speculation, recent studies have demonstrated that the exocyst complex regulates cell proliferation in multiple cell types. Bai et al. reported that downregulation of the exocyst complex inhibited head and neck cancer cell proliferation by reducing exosome secretion [32]. Coulter et al. reported that the atrophy and microcephaly observed in humans with EXOC7 variants is associated with loss of proliferating progenitor cells and post-mitotic neurons [33]. Nguyen et al. reported that deficiency of oocyte-specific Exoc1 impaired granulosa cell proliferation during follicle growth [34].

During kidney development Pax2 is expressed in the kidney tubules, including in PTCs [35], and regulates the transition of metanephric mesenchymal precursor cells into fully differentiated renal tubule epithelial cells in the early stages of embryogenesis [23, 36]. As development progresses, Pax2 expression in PTCs decline markedly after cells exit the mitotic cycle, indicating terminal differentiation [21]. It has been shown that aberrant Pax2 expression during kidney development disrupts normal nephrogenesis [23]. Moreover, recent studies have shown that Pax2 is re-expressed in the mature kidney under certain pathological conditions, including kidney fibrosis [24]. Maeshima et al. reported that Pax2 is re-expressed in injured tubular epithelial cells after kidney I/R injury and this Pax2 re-expression promotes conversion of tubule cells into progenitor-like cells with the potential to generate multiple cell types [24–26, 37]. Sako et al. reported that I/R-induced reactivation of Pax2 in the proximal tubule upregulates cyclin-dependent kinase 4 (CDK4), inhibiting proximal tubule regeneration and inducing fibrosis [38]. Furthermore, Pax2 overexpression induces EMT in renal tubule epithelial cells, whereas Pax2 silencing inhibits the progression of fibrosis by promoting early tubular repair [39, 40]. In addition, Lazzeri et al. reported that, during AKI, only Pax2-expressing tubular epithelial cells in injured kidneys actively undergo complete mitosis, thereby driving tubular regeneration [41]. In the present study, Pax2-expressing cells were more abundant in Exoc5^KO^ than in Exoc5^WT^ mice at 21 days following I/R, whereas the number of proliferating cells was lower. The number of Pax2- and BrdU-co-expressing cells was consistently lower in Exoc5^KO^ than in Exoc5^WT^ mice. In addition, Exoc5 downregulation increased Pax2 expression and accelerated TGF-β-induced EMT in HK-2 cells. However, Exoc5 does not directly bind Pax2 expression, as evaluated by Co-IP experiments, suggesting that Exoc5- and Pax2-associated repair or fibrosis of injured kidney tubule cells is unlikely to be regulated, at least in part, through a direct protein-protein interaction. Future studies, including transcriptomic and chromatin-based analyses, will be necessary to define the mechanisms by which Exoc5 regulates Pax2 activation or expression.

Epithelial-to-mesenchymal transition is a biological process in which epithelial cells with apical–basolateral polarity reverts to a nonpolarized mesenchymal state, characterized by enhanced migratory capacity, elevated resistance to apoptosis, and increased production of extracellular matrix (ECM) components [42, 43]. Damaged tubular cells lose epithelial polarity and acquire mesenchymal features while inappropriate differentiation delays tubular repair and promotes the progression of kidney fibrosis [44–46]. Recent studies have shown that exocyst complex components are involved either positively or negatively in the EMT processes depending on the component [47–50]. Lu et al. reported that Exoc7 participates in EMT in cancer cells through isoform switching; the abnormal Exoc7 isoform activates the expression of Snail and ZEB2, inducing transitions between epithelial and mesenchymal phenotypes [47]. Tanaka et al. reported that TGF-β treatment increases the expression of Exoc4, but not Exoc3 or Exoc7, and accelerates EMT by increasing the expression of N-cadherin and Smad3/4 through the regulation of CREB binding protein [48]. Qian et al. reported that knockdown of Exoc1 in A549 cells, human lung cancer cells, inhibits Akt phosphorylation, thereby preventing EMT following TGF-β treatment [50]. In the present study, Exoc5 knockout in PTCs of mice exacerbated kidney fibrosis following kidney I/R injury, and Exoc5 knockdown in HK-2 cells accelerated EMT following TGF-β treatment. Therefore, we propose that Exoc5 acts as an antifibrotic factor. This idea is supported by our previous studies showing that Exoc5 colocalizes with ZO-1 at the tight junction, the plasma membrane area that maintains epithelial polarity, and that Exoc5 transfection in renal epithelial cells promotes tubulogenesis and transport of basolateral proteins such as E-cadherin [11]. In addition, upregulation of Exoc5 maintains epithelial barrier integrity in cultured renal tubular epithelial cells and promotes recovery following injury [12, 13]. In the present study, Exoc5 expression was negatively correlated with post-I/R kidney function. Furthermore, Exoc5 expression was reduced in PTCs from patients with AKI and CKD. These findings indicate that Exoc5 plays an important role in maintaining kidney function and recovery from injury. Thus, Exoc5 is essential for maintaining renal tubular polarity and tubular repair following injury, suggesting that Exoc5 may represent a therapeutic target to inhibit the AKI-to-CKD transition.

In the previous study, we found that inducible Exoc5 knockout in PTCs using SLC34a1-CreERT2 mice increased susceptibility to kidney I/R injury compared with control groups. However, in the present study, we did not find a significant difference in kidney susceptibility between Exoc5^KO^ mice and Exoc5^WT^ mice when evaluated by PCr, BUN, and tubular damage score. We speculate that this difference may be due to the different ischemic time or the difference in Cre driver strains as the present study used PEPCK-Cre. Kusaba et al. reported that the recombination efficiency of SLC34a1-CreERT2 mice is lower in the S3 segment—the most vulnerable region of the kidney—than in the S1/2 segments of the proximal tubule [31]. In addition, inducible and constitutive knockout may produce divergent phenotypes because of compensatory regulation and differences in recombination timing. To clarify the basis of these differences, further studies evaluating Cre recombination efficiency, segment specificity, and knockout timing are needed.

In summary, our findings indicate that Exoc5 regulates PTC regeneration to facilitate kidney repair and may inhibit the progression of kidney fibrosis, suggesting that Exoc5 may represent a therapeutic target to inhibit the AKI-to-CKD transition.

## Materials and methods

### Generation of kidney PTC-specific *Exoc5* gene-deleted mice

Exoc5-floxed (Exoc5^f/f^) mice were generated as previously described [8]. Phosphoenolpyruvate carboxykinase promoter-driven Cre recombinase transgenic (PEPCK-Cre) mice were obtained from the University of Nebraska Medical Center [51]. PTC-specific Exoc5 conditional knockout mice were generated by crossing Exoc5^f/f^ mice with PEPCK-Cre mice and were designated as *Exoc5*^*f/f*^*;PEPCK-Cre* (Exoc5^KO^). Exoc5^f/f^ littermates were used as controls. The genotyping of the mice is depicted in Supplementary Fig. 1A, B.

### Animal experiments

Male mice (8–10 weeks old) weighing 22–25 g were used and were randomly assigned to each indicated time point (*n* = 4 per time point) in three independent experiments. Mice were housed in a specific pathogen-free facility with sterilized food, water, and bedding under a 12 h light/12 h dark cycle at 22 °C. To induce ischemia, mice were anesthetized with intraperitoneal pentobarbital (50 mg/kg; Hanlim Pharm, Gyeonggi-do, Korea). Kidneys were exposed through flank incisions, and then renal pedicles were completely clamped for 25 min using microaneurysm clamps. For sham operations, the same procedure was performed without clamping the renal pedicle. Body temperature was maintained at 36.4–37 °C during surgery using a surgical heating pad. Some mice were administered 5’-bromo-2’-deoxyuridine (BrdU; 50 mg/kg; Sigma-Aldrich, St. Louis, MO, United States) intraperitoneally. Mice were euthanized with an overdose of pentobarbital sodium at 1, 7 and 21 days after surgery. Kidneys were collected by snap-freezing in liquid nitrogen for biochemical analysis or by perfusion fixation in PLP (4% paraformaldehyde, 75 mM L-lysine, 10 mM sodium periodate) for histological studies.

### Blood and urine biochemistry

To assess kidney function, blood was collected using a heparinized syringe, and urine was collected in metabolic cages. Plasma creatinine (PCr), blood urea nitrogen (BUN), and urine creatinine were measured using a Vitro 250 Chemistry Analyzer (Johnson & Johnson, New Brunswick, NJ, United States). Glomerular filtration rate (GFR) was calculated.

### Western blot analysis

Western blot analysis was performed as previously described [52]. Antibodies against the following proteins were used: Exoc5 (Cat no. sc-514802, Santa Cruz, CA, United States), alpha-smooth muscle actin (α-SMA; Cat no. A2547, Sigma-Aldrich), Vimentin (Cat no. #5741, Cell Signaling Technology), PCNA (Cat no. m879, DAKO, Carpinteria, CA, USA), p-ERK (Cat no. #9101, Cell Signaling Technology, Danvers, MA, United States), t-ERK (Cat no. #4695, Cell Signaling Technology), Pax2 (Cat no. ab79389, Abcam, Cambridge, UK), N-cadherin (Cat no. #13116, Cell Signaling Technology), phosphorylated-Smad3 (p-Smad; Cat no. ab52903, Abcam), and glyceraldehyde-3-phosphate dehydrogenase (GAPDH; Cat no. NBP600-502, NOVUS, Littleton, CO, USA).

### Immunofluorescence staining

Following deparaffinization, sections were incubated in PBS containing 0.2% Triton X-100 (Sigma-Aldrich) for 1 min and washed in PBS for 10 min. For antigen retrieval, sections were boiled in 10 mM sodium citrate buffer (pH 6.0) for 10 min, cooled for 20 min, and washed 3 times with PBS for 5 min per wash. Sections were blocked in PBS containing 3% bovine serum albumin (blocking buffer) for 30 min and then incubated with antibodies against Exoc5 (Cat no. 17593-1-AP, Proteintech, Rosemont, IL, USA), AQP1 (Cat no. MCA2099, Bio-Rad Laboratories, CA, USA), AQP1 (Cat no. ab168387, Abcam), α-SMA (Cat no. A2547, Sigma-Aldrich), Vimentin (Cat no. #5741, Cell Signaling Technology), E-cadherin (Cat no. #3195, Cell Signaling Technology), BrdU (Cat no. ab6326, Abcam), and Pax2 (Cat no. PRB-276P, Covance, North Carolina, United States) at 4 °C overnight. After washing, sections were incubated with FITC-, Texas Red-, or Cy3-conjugated goat anti-rabbit or anti-mouse IgG for 60 min and then washed 3 times with PBS for 5 min per wash. Nuclei were stained with 4′,6-diamidino-2-phenylindole (DAPI; Sigma-Aldrich). Sections were observed under a Leica microscope (DM2500).

### Periodic acid–Schiff (PAS) staining

Kidney sections were stained with PAS as described previously [53]. Kidney damage was scored in a blinded manner, and the slides were independently evaluated by three investigators.

### Picrosirius red staining

Kidney sections were fixed with Bouin’s solution at 55 °C for 1 h, washed in running tap water until the yellow coloration disappeared, incubated in 0.1% fast green (Fisher Scientific, Lagoas Park, Portugal) for 10 min, and washed with 0.5% acetic acid. Sections were then stained with Picrosirius red for 1 h.

### Single-nucleus RNA (snRNA-seq) sequencing data analysis

snRNA-seq data of the kidneys from patients with AKI and CKD were obtained from the Gene Expression Omnibus (GEO) database (GSE210622 for AKI and GSE199711 for CKD). AKI kidney samples consisted of postmortem biopsies from eight brain-dead donors diagnosed with AKI within 5 days (*n* = 8), and control samples included tumor-adjacent normal kidney tissues and postmortem biopsies obtained from a brain-dead donor at different time points (*n* = 6). CKD kidney samples consisted of nephrectomy specimens from three patients with diabetic or hypertensive CKD (*n* = 3), and control samples consisted of nephrectomy kidney tissues from two individuals without CKD (*n* = 2). All datasets were analyzed using the Seurat R package (v4.5.2) [54, 55]. Seurat objects were generated for each dataset, and nuclei with fewer than 500 detected genes or more than 5% of mitochondrial gene expression were excluded. After normalization and clustering, 22 clusters in the AKI dataset and 18 clusters in the CKD dataset were identified and annotated based on cell-type marker genes (Supplementary Fig. 2A, B). Gene expression analyses were subsequently performed in proximal tubule cell clusters.

### Cell culture

HK-2 cells (ATCC, Manassas, VA, USA) were cultured in Dulbecco’s Modified Eagle Medium: Nutrient Mixture F12 (DMEM/F12) (Thermo Fisher Scientific, Waltham, MA, United States) containing 5% fetal bovine serum (Thermo Fisher Scientific) and 100 U/mL streptomycin/penicillin (S/P) (WelGENE Inc., Daegu, Korea) at 37 °C in a humidified atmosphere containing 5% CO_2_. At 70%–80% confluence, HK-2 cells were incubated in Opti-MEM (Thermo Fisher Scientific) without S/P for 2 h and transfected with 25 or 50 nmol/L of small interfering RNA (siRNA) targeting Exoc5 (sense: 5′-GCA CAU UAG CUA UGU AGC AAC UAA A-3′, antisense: 5′-UUU AGU UGC UAC AUA GCU AAU GUG C, Bioneer, Daejeon, Korea) and Pax2 (sense: 5′-CUG AAG UUG AGU UUG AGA-3′, antisense: 5′-CUC UCA AAC UCA ACU UCA G, Bioneer) or scrambled siRNA (Cat no. SN-1002, Bioneer) using Lipofectamine 3000 (Thermo Fisher Scientific) for 7 h according to the manufacturer’s instructions. Medium was replaced with DMEM/F12, and cells were treated with 5 ng/mL TGF-β (R&D Systems, Minneapolis, MN, USA) or vehicle for 48 h.

### Co-immunoprecipitation (Co-IP)

Co-IP was performed using the Pierce Co-Immunoprecipitation kit (Cat no. 26149, Thermo Fisher Scientific) according to the manufacturer’s protocol. Briefly, the an anti-Exoc5 (Cat no. sc-514802, Santa Cruz) antibody and mouse normal IgG (Cat no. #12-371, Sigma-Aldrich) were immobilized on the coupling resin, and whole-cell lysates of HK-2 cells were incubated with the antibody-resin complex at 4 °C. After washing, bound proteins were eluted and further analyzed by western blot.

### Statistics

Data were analyzed using GraphPad Prism 6 (GraphPad, San Diego, CA, USA). Data are presented as mean ± SEM. Statistical analysis was performed using Student’s *t*-test and one-way analysis of variance with Tukey’s post hoc procedure. Statistical significance was set at *p* < 0.05.

## Supplementary information

Supplemental figure. 1-3

Uncropped western blot

## Data Availability

All datasets generated for this study are included in the article/Supplementary figure.
