# Supplementary material for: Deletion of exocyst component 5 suppresses repair of injured kidney by limiting cell proliferation
Source: Cell Death Discov. 2026 Apr 24;12:269. doi: 10.1038/s41420-026-03127-6 (PMC13237231; doi:10.1038/s41420-026-03127-6)
Supplement: Supplementary file 1 — Supplemental figure. 1-3 [file 41420_2026_3127_MOESM1_ESM.pdf]

Supplemental Fig. 1A, B

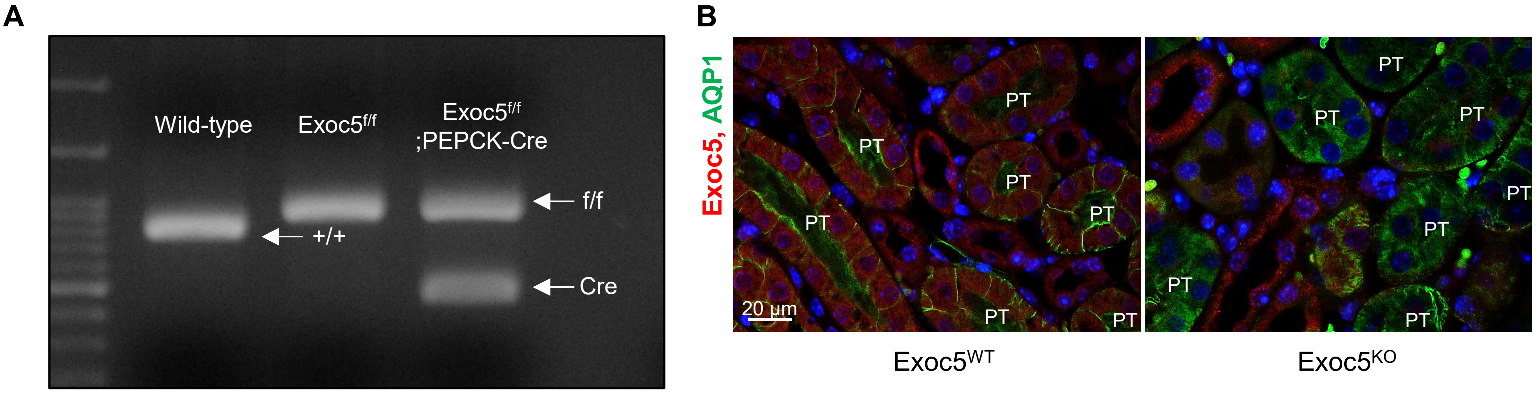

Supplemental Figure 1. Validation of Exoc5 knockout (A) Mice genotypes were determined by PCR from tail genomic DNA using the following primers: Exoc5 loxP (Forward: 5'-GCCTGTAACTCACAGAGATC-3'. Reverse: 5'-GCTGGCATTCTAAGTCATGG-3') and PEPCK Cre (Forward: 5'-CGGTGCTAACCAGCGTTTTC-3'. Reverse: 5'-TGGGCGGCATGGTGCAAGTT-3'). (B) Kidney sections were immunofluorescence-stained with antibodies against Exoc5 (red) and AQP1(green); DAPI (blue) was used to visualize nuclei. PT: proximal tubule.

Supplemental Fig. 2A, B

A

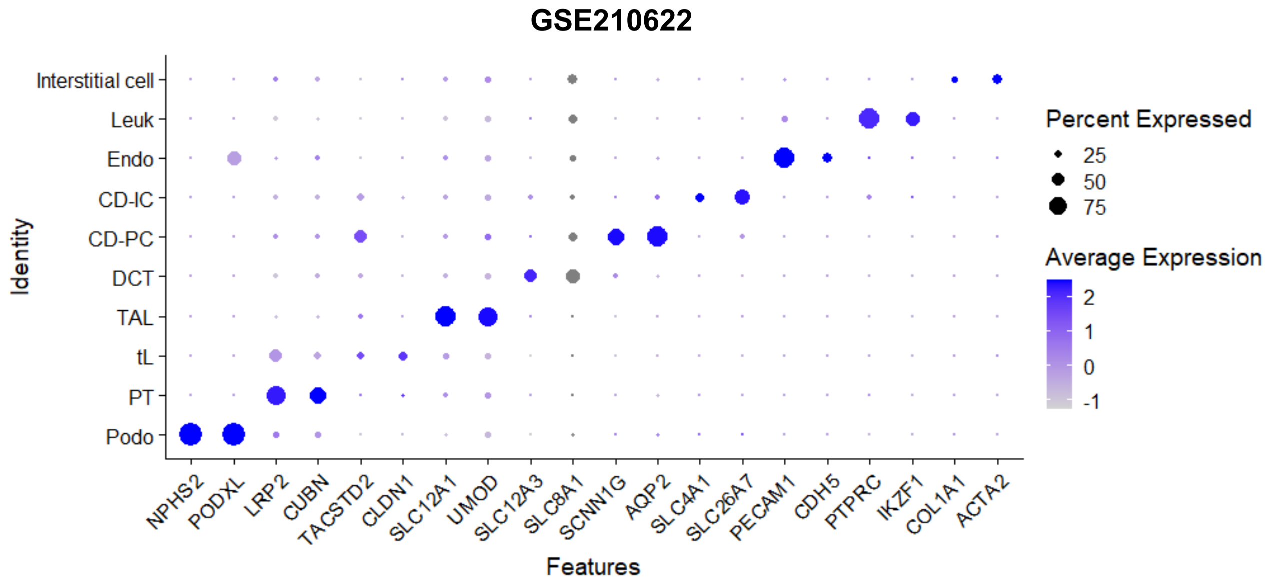

B

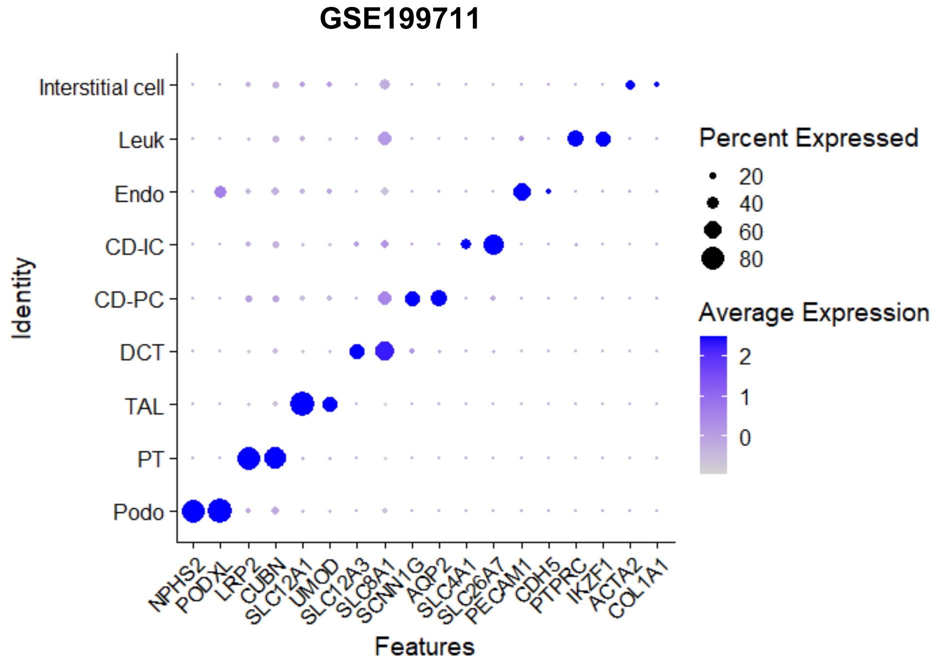

Supplemental Figure 2. Marker gene expression used for cell type annotation in the AKI and CKD snRNA-seq datasets (A, B) Dot plot showing the expression of marker genes used to assign major kidney cell types in the AKI and CKD datasets. Major cell types of the human kidney (Podo, podocytes; PT, proximal tubule; tL, thin limb; TAL, thick ascending limb; DCT, distal convoluted tubule; CD-PC/IC, collectind duct principal/intercalated cells; Endo, endothelial cells; Leuk, leukocytes)

Supplemental Fig. 3A-F

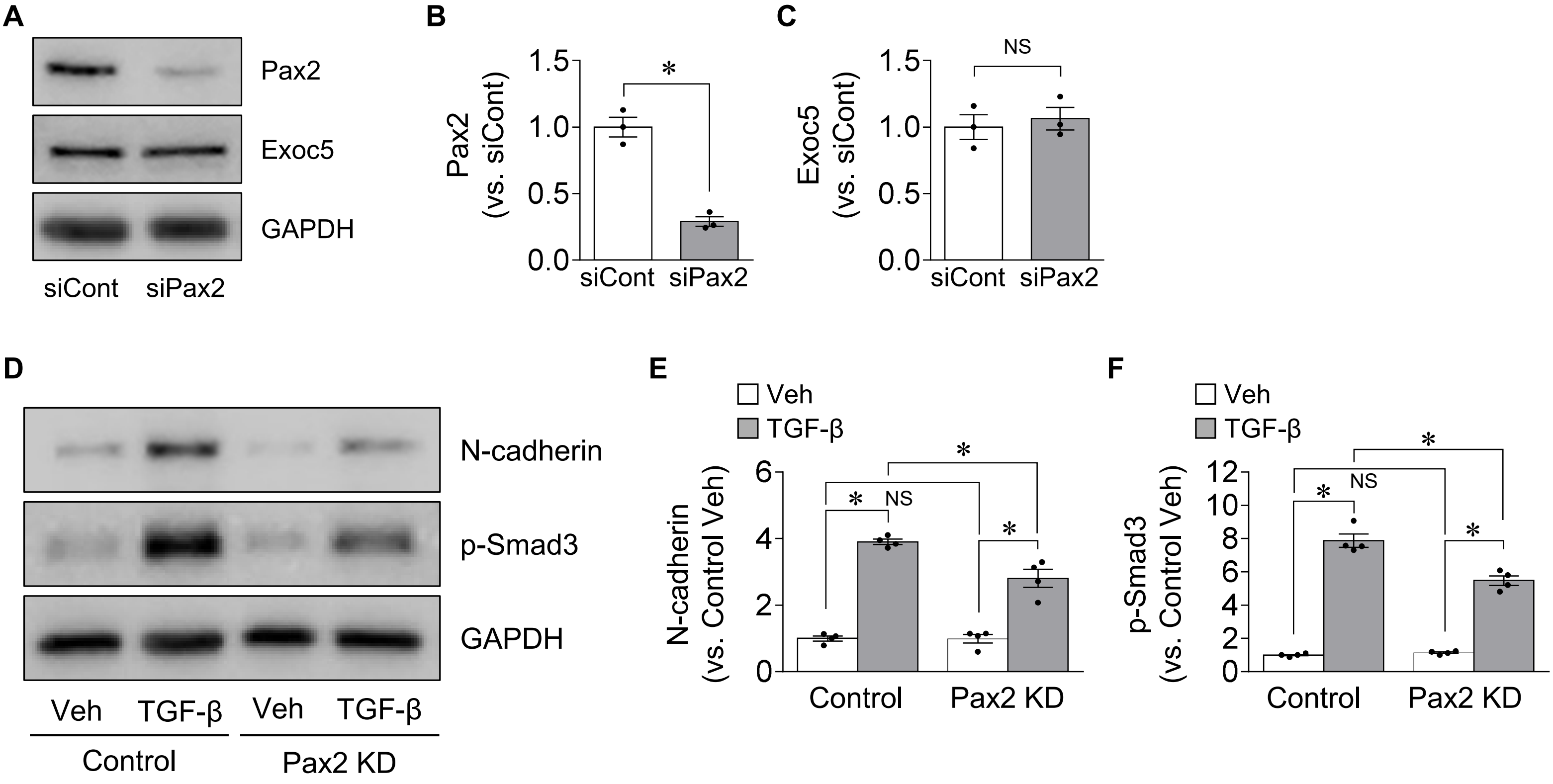

Supplemental Figure 3. N-cadherin and p-Smad3 expression in Pax2-siRNA-treated HK-2 cells after TGF-β treatment HK-2 cells were transfected with either Pax2-siRNA (50 nmol/L) or scrambled siRNA (siCont, 50 nmol/L) and then treated with or without 5 ng/mL TGF-β for 48 h. (A–F) Pax2, Exoc5, N-cadherin and p-Smad3 levels in cell lysates were analyzed by Western blot, with GAPDH as the loading control; band densities were quantified using ImageJ. Results are expressed as mean ± SEM (n = 3–4). *, $p < 0.05$; NS, not significant.
