# Supplementary material for: Deletion of exocyst component 5 suppresses repair of injured kidney by limiting cell proliferation
Source: Cell Death Discov. 2026 Apr 24;12:269. doi: 10.1038/s41420-026-03127-6 (PMC13237231; doi:10.1038/s41420-026-03127-6)

Uncropped Western blot

Fig.1F

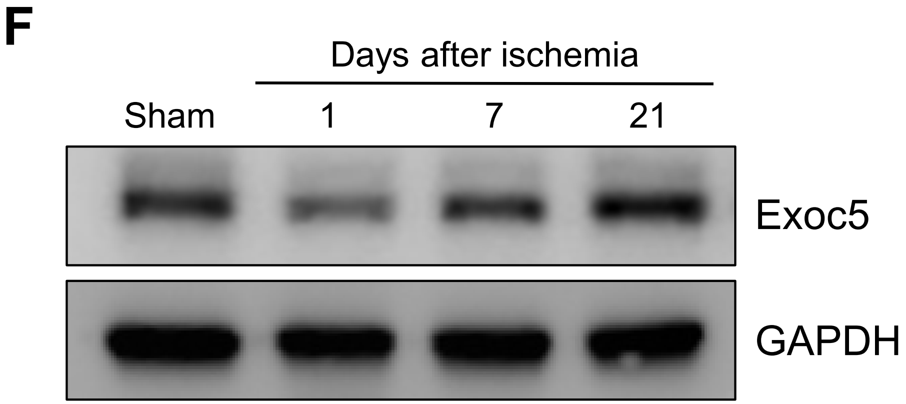

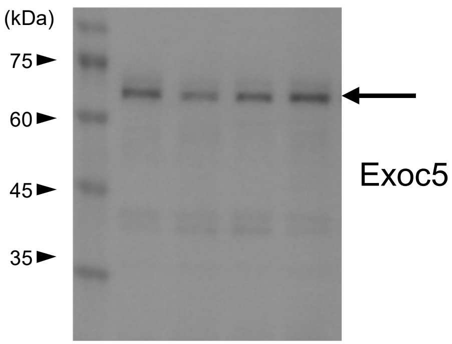

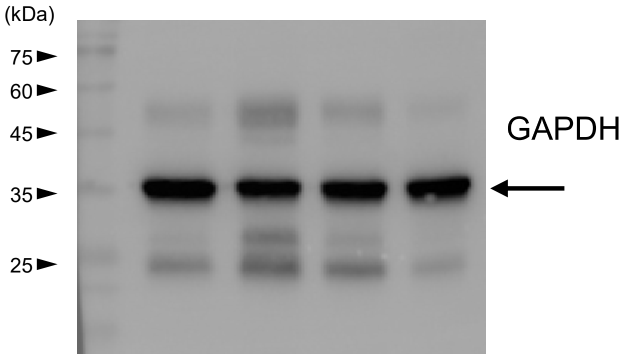

Uncropped Western blot

Fig.3G

G

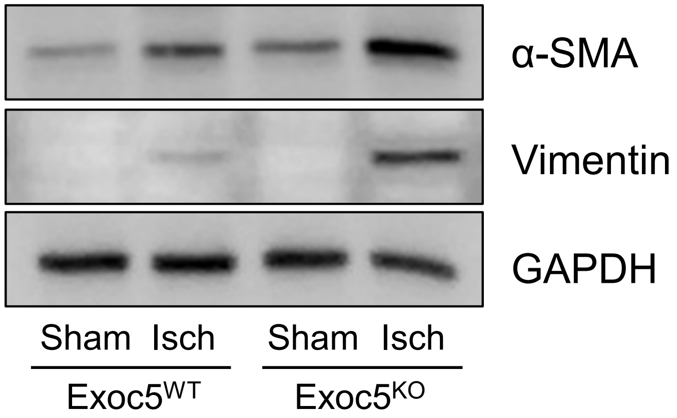

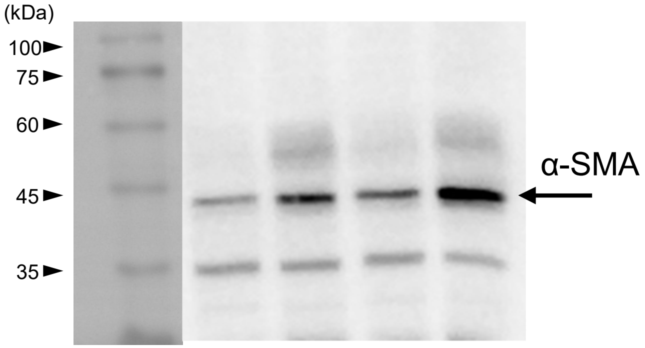

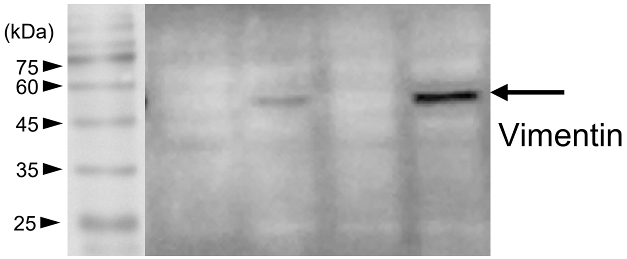

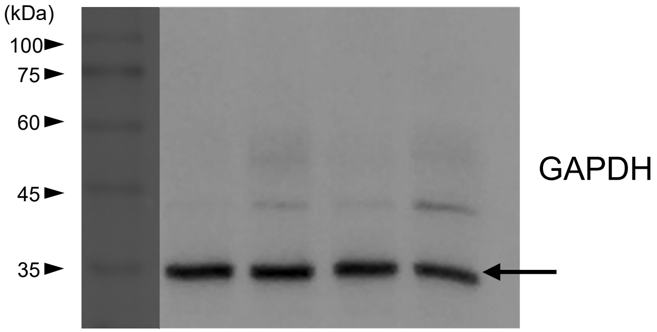

Uncropped Western blot

Fig.4C

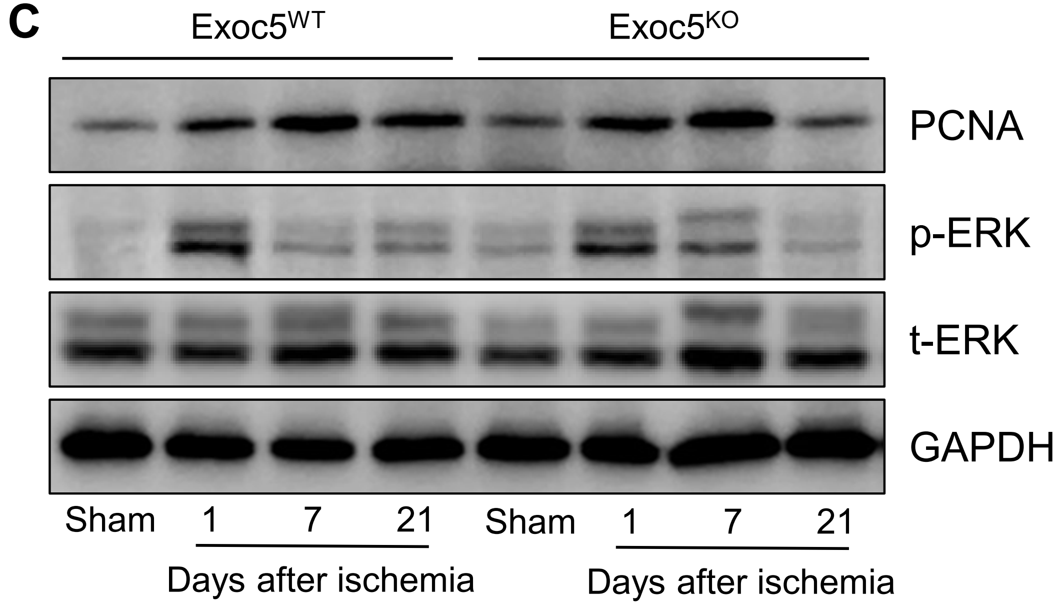

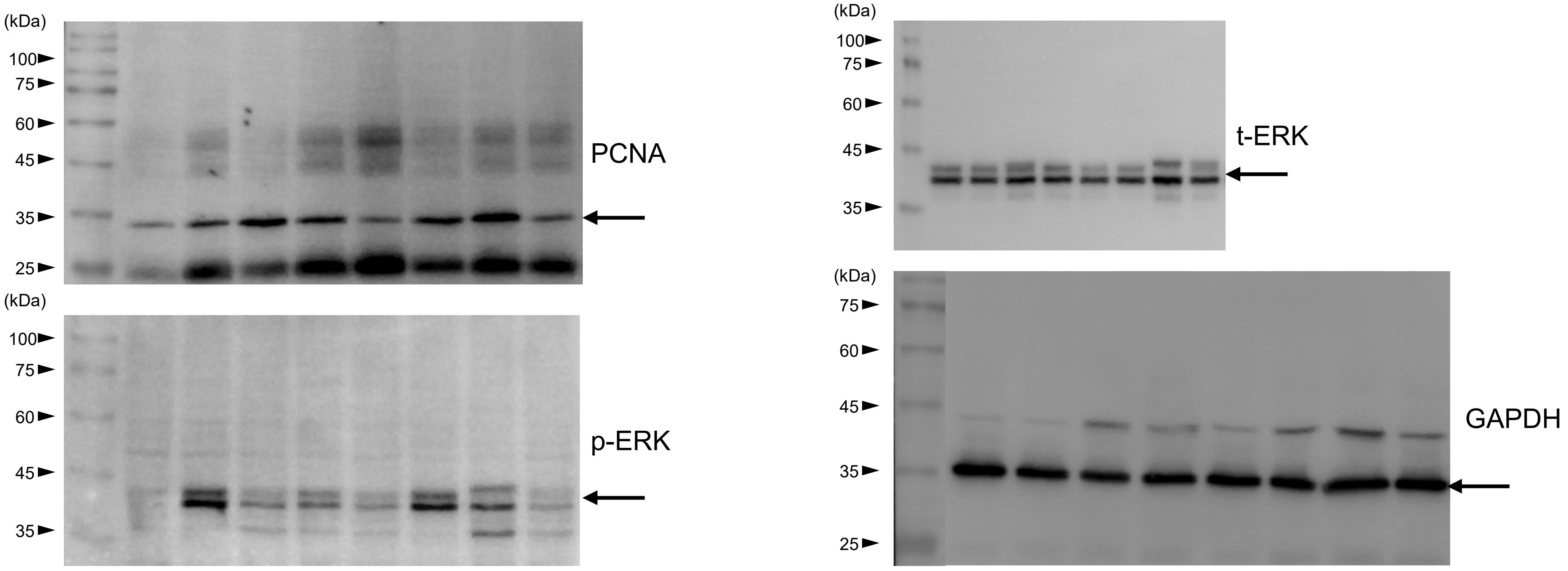

Uncropped Western blot

Fig.5A

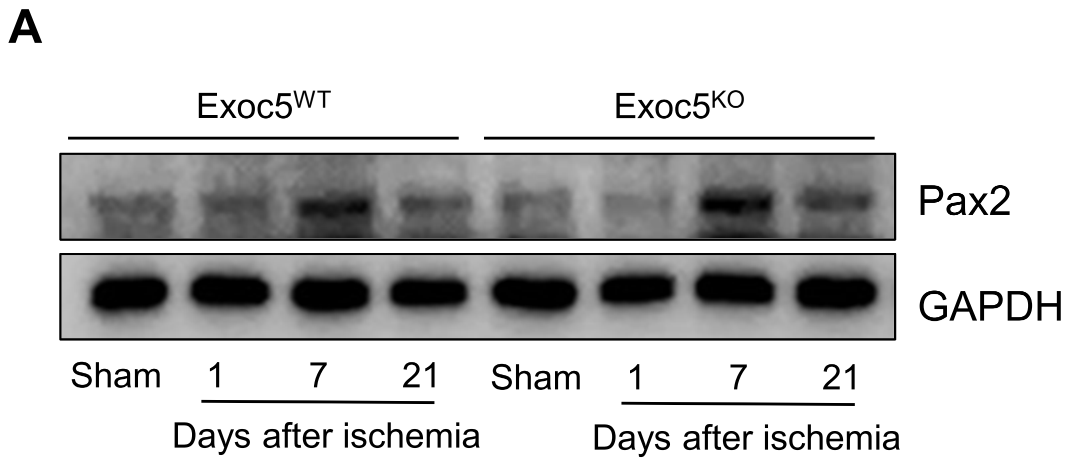

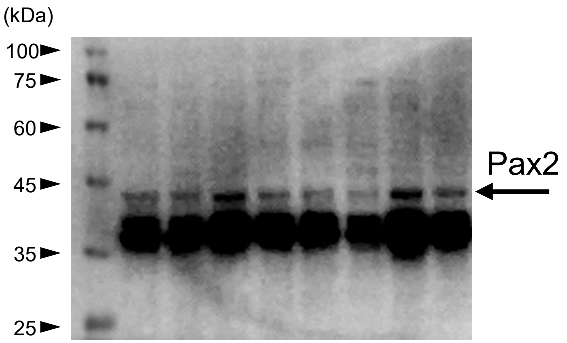

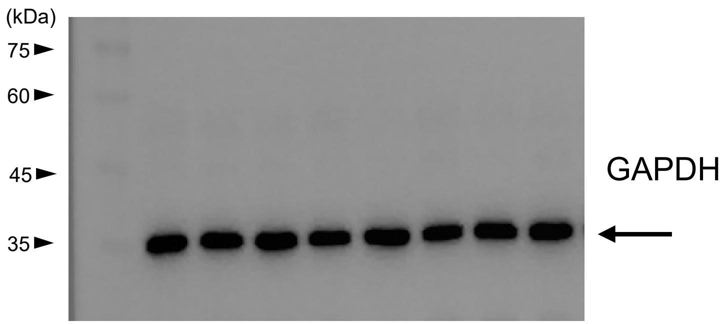

Uncropped Western blot

Fig.6A

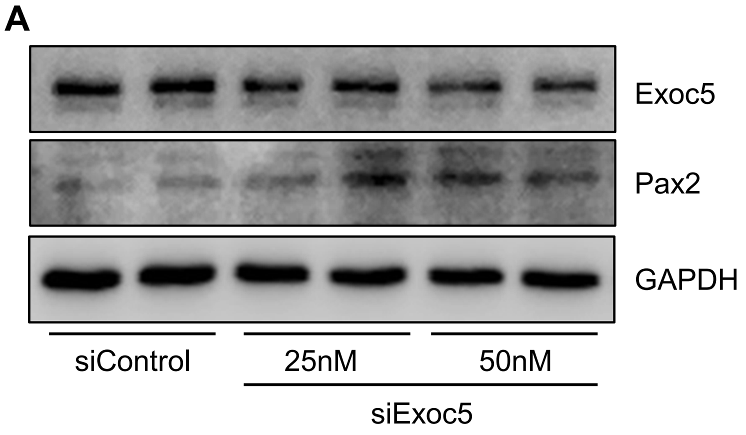

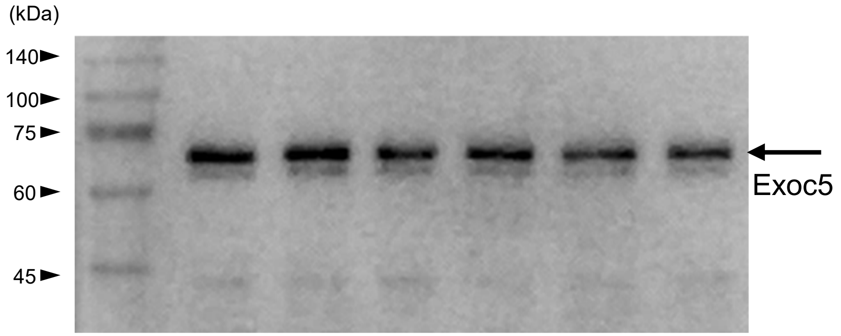

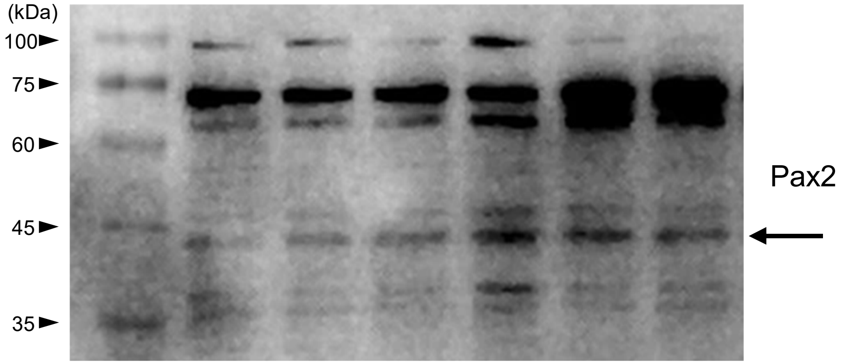

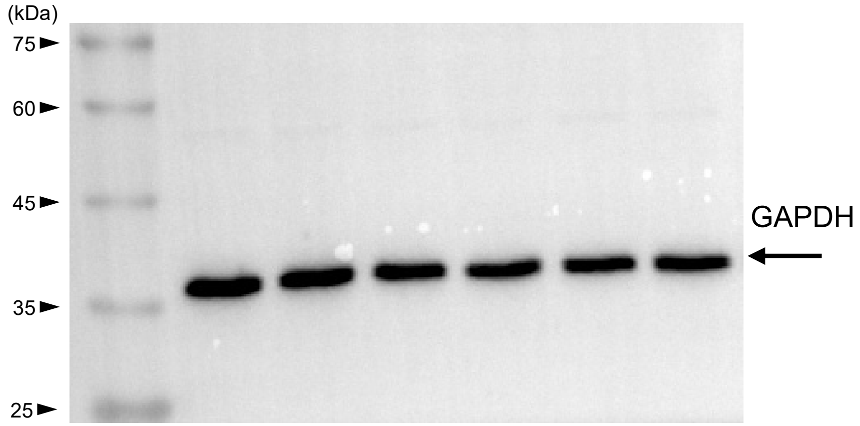

Uncropped Western blot

Fig.6D

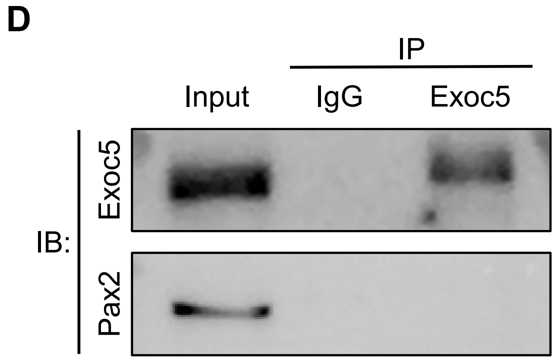

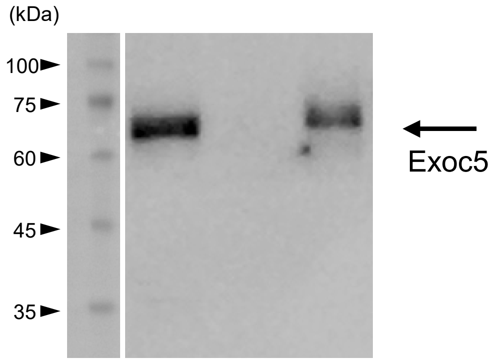

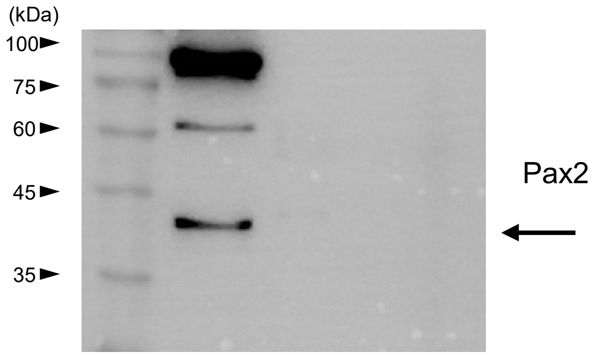

Uncropped Western blot

Fig.6E

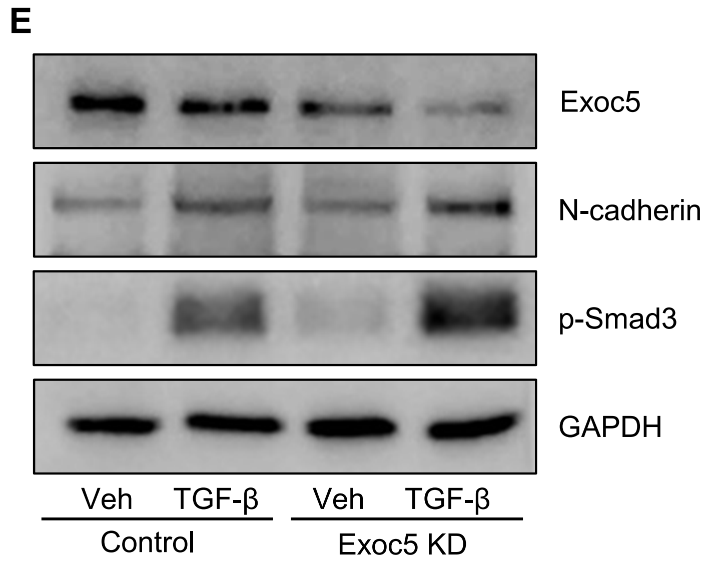

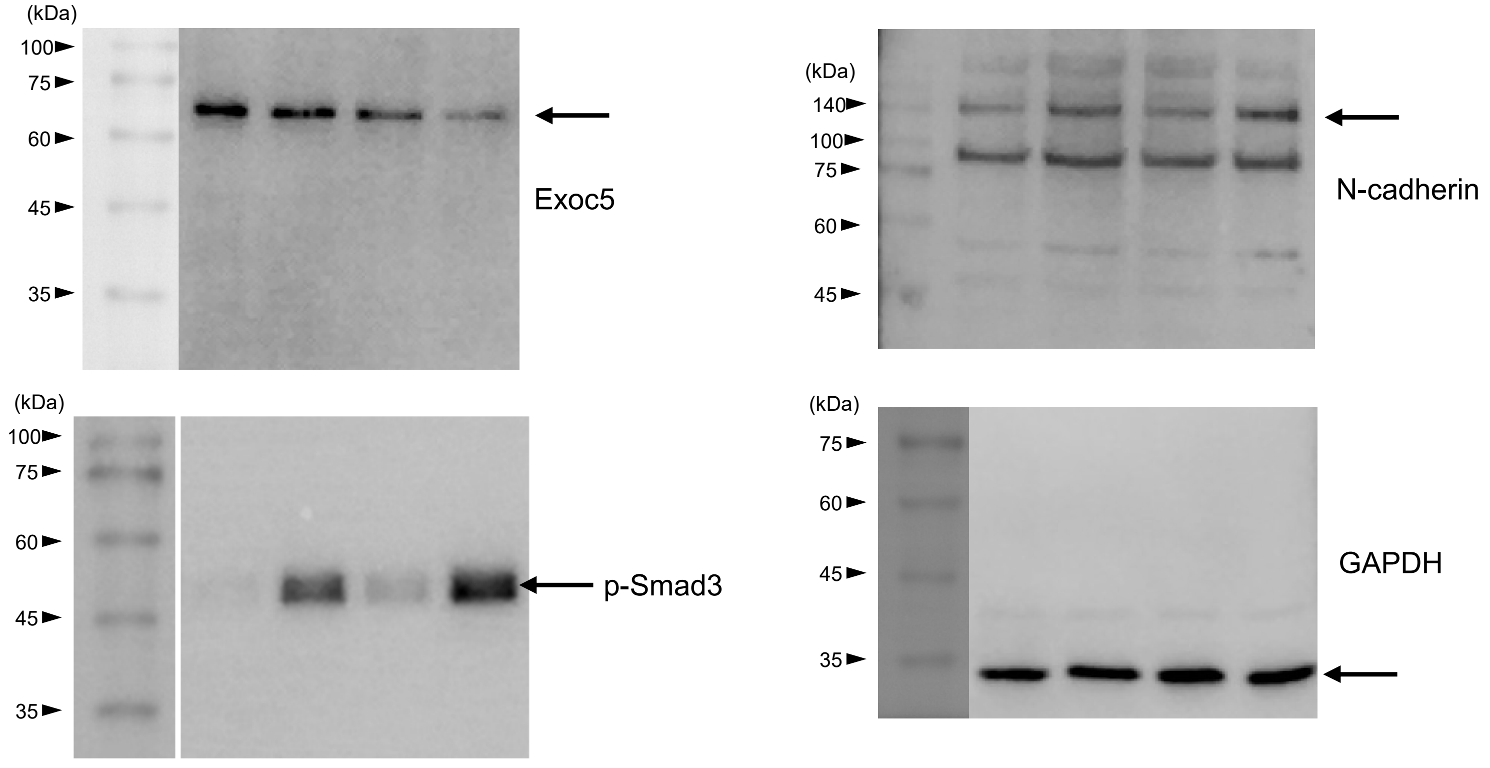

Uncropped Western blot

Supplemental Fig.3A

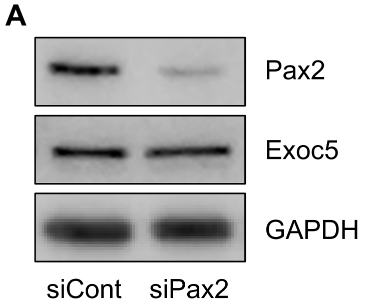

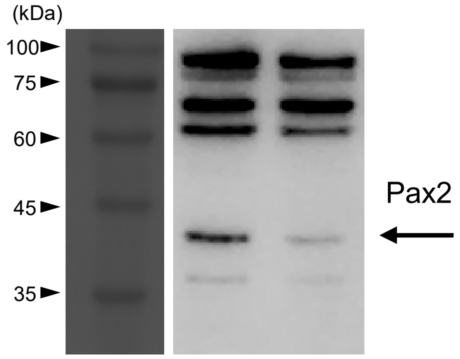

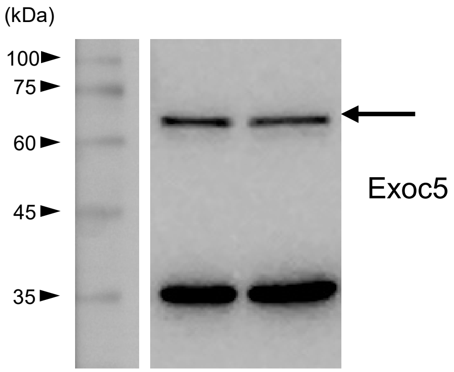

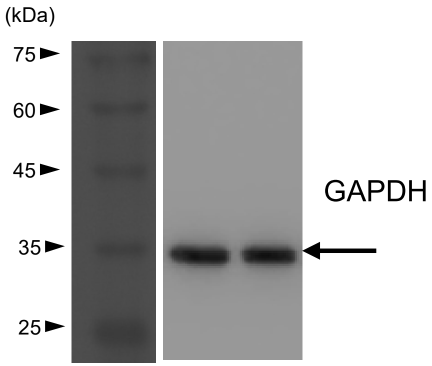

Uncropped Western blot

Supplemental Fig.3D

D

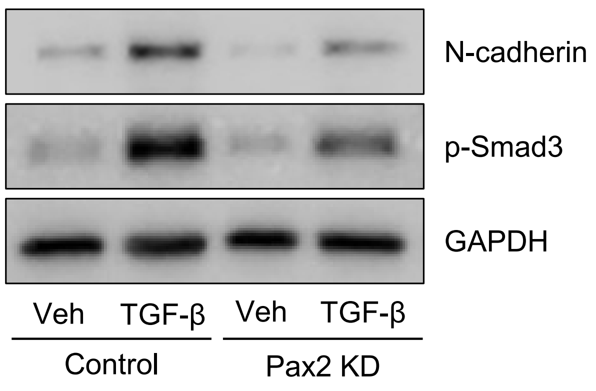

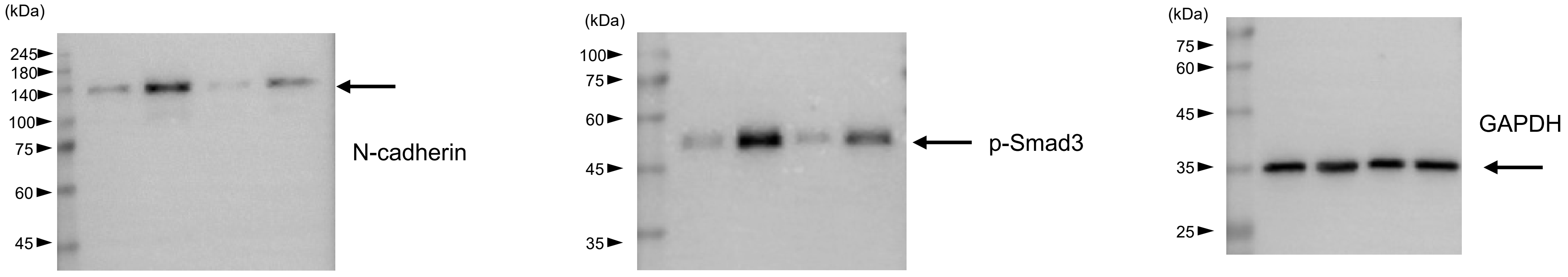

Supplement: Supplementary file 2 — Uncropped western blot [file 41420_2026_3127_MOESM2_ESM.pdf]
